# Tuberculous Peripancreatic Lymphadenitis Presenting as Obstructive Jaundice Due to Extrinsic Common Bile Duct Compression: A Case Report

**DOI:** 10.7759/cureus.104959

**Published:** 2026-03-09

**Authors:** Bassem Al Hariri, Abdulqadir J Nashwan

**Affiliations:** 1 Medicine, Qatar University, Doha, QAT; 2 Medicine, Weill Cornell Medicine-Qatar, Doha, QAT; 3 Internal Medicine, Hamad Medical Corporation, Doha, QAT; 4 Nursing and Midwifery Research, Hamad Medical Corporation, Doha, QAT

**Keywords:** common bile duct compression, eus-fnb, extra biliary tuberculosis, mrcp, obstructive jaundice, tuberculous lymphadenitis

## Abstract

We report the case of a 29-year-old male from the Indian subcontinent (a tuberculosis (TB)-endemic region) residing in Qatar, who presented with a three-day history of epigastric pain, dark urine, and pale stools. He had no known TB exposure, was HIV-negative, and had received the Bacillus Calmette-Guérin or BCG vaccination in childhood. Physical examination revealed scleral icterus without peripheral lymphadenopathy. Initial imaging showed a dilated common bile duct (CBD) (10 mm) and an overdistended gallbladder with sludge. Magnetic resonance imaging demonstrated conglomerated necrotic lymphadenopathy in the portacaval and precaval regions (the largest measuring 44 × 25 mm), causing smooth extrinsic compression of the distal CBD. Contrast-enhanced CT confirmed central necrosis with peripheral rim enhancement, favoring a tuberculous etiology over a malignant one. Endoscopic ultrasound-guided fine needle biopsy of the peripancreatic lymph nodes revealed necrotizing granulomatous inflammation with caseous necrosis. Ziehl-Neelsen staining for acid-fast bacilli was negative, which can occur in paucibacillary TB. Xpert MTB/RIF Ultra assay detected *Mycobacterium tuberculosis* complex DNA without rifampicin resistance mutations. Mycobacterial culture was sent and remains pending. The patient was HIV-negative, and QuantiFERON-TB Gold Plus was positive. After consultation with the infectious diseases team, he started a hepatoprotective anti-tubercular regimen (moxifloxacin, ethambutol, amikacin, and linezolid with pyridoxine) due to significant baseline hepatocellular injury (alanine aminotransferase peaking at 465 U/L), resulting in significant clinical and biochemical improvement. At three- and six-month follow-up, liver function tests normalized, and repeat imaging showed near-complete resolution of lymphadenopathy with no residual biliary dilatation. He was discharged on therapy with close outpatient follow-up. This case highlights the importance of considering tuberculous peripancreatic lymphadenitis in the differential diagnosis of obstructive jaundice, particularly in young patients from TB-endemic regions, and underscores the critical role of tissue acquisition for definitive diagnosis.

## Introduction

Obstructive jaundice is most frequently caused by choledocholithiasis (accounting for 50%-60% of cases), pancreatic malignancies (15%-20%), or benign biliary strictures (10%-15%) [1]. The mechanism of obstructive jaundice involves mechanical interruption of bile flow from the liver to the duodenum, which can occur at any level from the intrahepatic bile ducts to the ampulla of Vater. Extrinsic compression of the common bile duct (CBD) can result from enlarged lymph nodes in the porta hepatis, portacaval region, or peripancreatic area. Common causes of significant lymphadenopathy in this region include metastatic malignancy (particularly from gastrointestinal primaries), lymphoma, and infectious etiologies such as tuberculosis (TB) [2].

While TB remains a significant global health burden, abdominal TB accounts for approximately 11%-12% of extrapulmonary cases and can involve the gastrointestinal tract (75%), peritoneum (60%), lymph nodes (25%-50%), and solid organs (15%) [3]. The pathophysiology of abdominal TB typically involves hematogenous spread from a primary pulmonary focus or ingestion of mycobacteria, leading to latent infection that may reactivate years later [4].

Hepatobiliary TB represents a rare subset of abdominal TB, encompassing hepatic parenchymal disease (micronodular or macronodular TB, tuberculoma, or abscess formation) and biliary involvement. Biliary obstruction due to TB is exceedingly rare and typically results from extrinsic compression by enlarged caseous lymph nodes rather than intrinsic biliary involvement [5].

Obstructive jaundice secondary to tuberculous lymphadenopathy is an exceptionally rare manifestation, with limited cases documented in the literature [6-8]. The pathophysiologic mechanism involves caseous necrosis and progressive enlargement of periportal, peripancreatic, or portacaval lymph nodes, which can externally impinge upon the adjacent CBD [7]. The non-specific clinical and radiological presentation of this condition often leads to a misdiagnosis of malignancy (particularly pancreatic cancer or lymphoma), resulting in delayed treatment and potentially unnecessary surgical interventions [8]. Previous case reports have described similar presentations where tuberculous lymphadenitis masqueraded as pancreatic malignancy, highlighting the diagnostic challenge this condition poses [5-7]. We present a case of obstructive jaundice caused by peripancreatic tuberculous lymphadenitis in a young patient from a TB-endemic region, illustrating a methodical diagnostic approach that leveraged advanced imaging and tissue acquisition to secure the diagnosis and guide appropriate medical therapy, thereby avoiding unnecessary surgical intervention.

## Case presentation

A 29-year-old male originating from the Indian subcontinent (a region with a high incidence of TB) and currently residing in Qatar for the past three years presented to the emergency department with a three-day history of worsening epigastric and right upper quadrant pain. The pain was exacerbated by food intake and associated with dyspepsia and self-induced vomiting. He also reported passing dark urine for three days and one episode of pale stool. He denied any fever, pruritus, or constitutional symptoms such as night sweats or weight loss. He had no known sick contacts, no family history of TB, and no history of travel outside Qatar in the past two years. He was a non-smoker and consumed alcohol only occasionally (approximately one to two units per month). He worked as a construction worker and lived with his family in shared accommodation. He had received the Bacillus Calmette-Guérin or BCG vaccination as a child. He had no history of diabetes mellitus, immunosuppressive medication use, or previous TB treatment.

On physical examination, his vital signs were: temperature 36.8°C, heart rate 78 beats per minute, blood pressure 125/78 mmHg, respiratory rate 16 breaths per minute, and oxygen saturation 98% on room air. He was alert and oriented, in no acute distress. He was noted to have scleral icterus. No conjunctival pallor, jugular venous distention, or peripheral lymphadenopathy (cervical, supraclavicular, axillary, or inguinal) was present. Cardiovascular examination revealed regular rate and rhythm with no murmurs, rubs, or gallops. Respiratory examination demonstrated clear breath sounds bilaterally without wheezes or crackles. Abdominal examination revealed mild tenderness in the epigastrium and right upper quadrant, without guarding or rigidity. Murphy's sign was negative. No palpable masses, organomegaly, or hepatosplenomegaly were noted. Bowel sounds were normal. Skin examination showed no rash, though jaundice was noted. Body mass index was 22.4 kg/m² (within the normal range).

Initial laboratory investigations revealed a mixed cholestatic and hepatocellular injury pattern. Comprehensive laboratory testing is presented in Table 1.

**Table 1:** Initial laboratory investigations Bilirubin values are reported in μmol/L (conversion factor: 1 mg/dL = 17.1 μmol/L).

Laboratory test	Result	Reference range
Hematology		
Hemoglobin	13.8 g/dL	13.0-17.0 g/dL
White blood cell count	7.2 × 10³/μL	4.0-10.0 × 10³/μL
Neutrophils	65%	40%-75%
Lymphocytes	28%	20%-45%
Monocytes	5%	2%-10%
Eosinophils	1.50%	0.5%-5.0%
Basophils	0.50%	0%-1.0%
Platelets	289 × 10³/μL	150-400 × 10³/μL
Liver function tests		
Total bilirubin	51 μmol/L	<21 μmol/L
Direct bilirubin	48 μmol/L	<3.4 μmol/L
Alkaline phosphatase (ALP)	317 U/L	40-130 U/L
Alanine aminotransferase (ALT)	244 U/L	0-41 U/L
Aspartate aminotransferase (AST)	194 U/L	0-40 U/L
Gamma-glutamyl transferase (GGT)	156 U/L	10-71 U/L
Total protein	68 g/L	60-80 g/L
Albumin	38 g/L	35-50 g/L
Renal function and electrolytes		
Sodium	138 mmol/L	135-145 mmol/L
Potassium	4.2 mmol/L	3.5-5.1 mmol/L
Chloride	101 mmol/L	98-107 mmol/L
Calcium	2.35 mmol/L	2.15-2.55 mmol/L
Phosphorus	1.1 mmol/L	0.8-1.5 mmol/L
Blood urea nitrogen (BUN)	5.2 mmol/L	2.5-7.8 mmol/L
Creatinine	78 μmol/L	62-106 μmol/L
Pancreatic enzymes		
Lipase	77 U/L	13-60 U/L
Amylase	68 U/L	28-100 U/L
Inflammatory markers		
C-reactive protein (CRP)	8.1 mg/L	<5.0 mg/L
Erythrocyte sedimentation rate (ESR)	22 mm/hr	<15 mm/hr
Coagulation profile		
International normalized ratio (INR)	1.1	0.9-1.2
Autoimmune markers		
Antinuclear antibody (ANA)	Negative	Negative
Anti-mitochondrial antibody (AMA)	Negative	Negative
Anti-smooth muscle antibody (ASMA)	Negative	Negative
Liver-kidney microsomal antibody (LKM-1)	Negative	Negative

**Figure 1:**
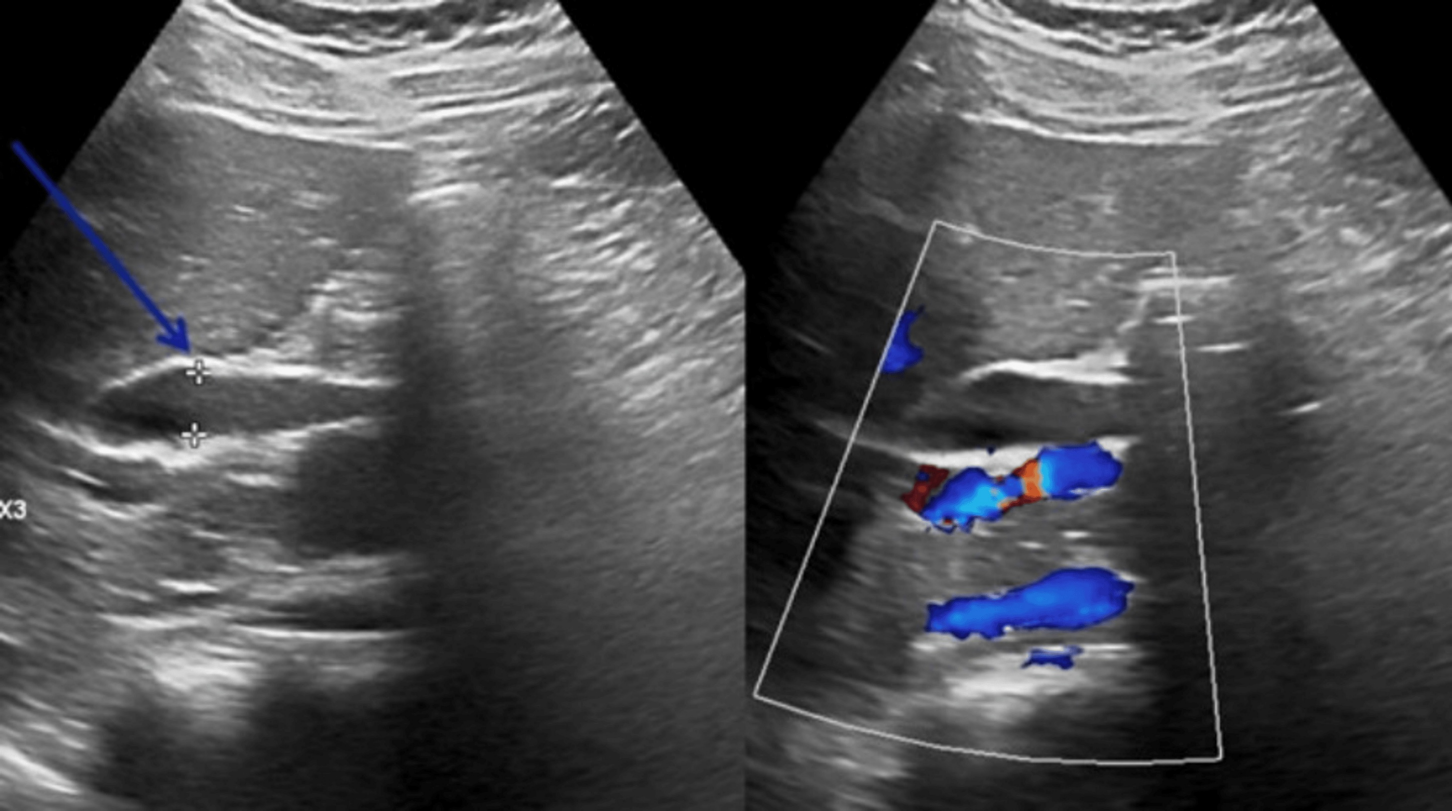
Abdominal ultrasound A transverse image demonstrating a dilated common bile duct, measuring 10 mm in diameter (indicated by calipers). This finding of biliary dilatation without evidence of choledocholithiasis prompted further cross-sectional imaging. The gallbladder and peripancreatic region are not well visualized in this image due to technical limitations and bowel gas interference.

An abdominal ultrasound demonstrated a dilated CBD measuring 10 mm, an overdistended gallbladder measuring 11.5 × 4.2 cm with sludge layering dependently, and a 7.6 mm sessile polyp along the posterior wall; no gallstones or wall thickening were identified. The intrahepatic biliary radicals were mildly prominent. The pancreas appeared normal without a mass or a focal lesion. The visualized portions of the liver were homogeneous without focal lesions. Peripancreatic and portacaval lymph nodes were not clearly visualized due to bowel gas (Figure 1).

**Figure 2:**
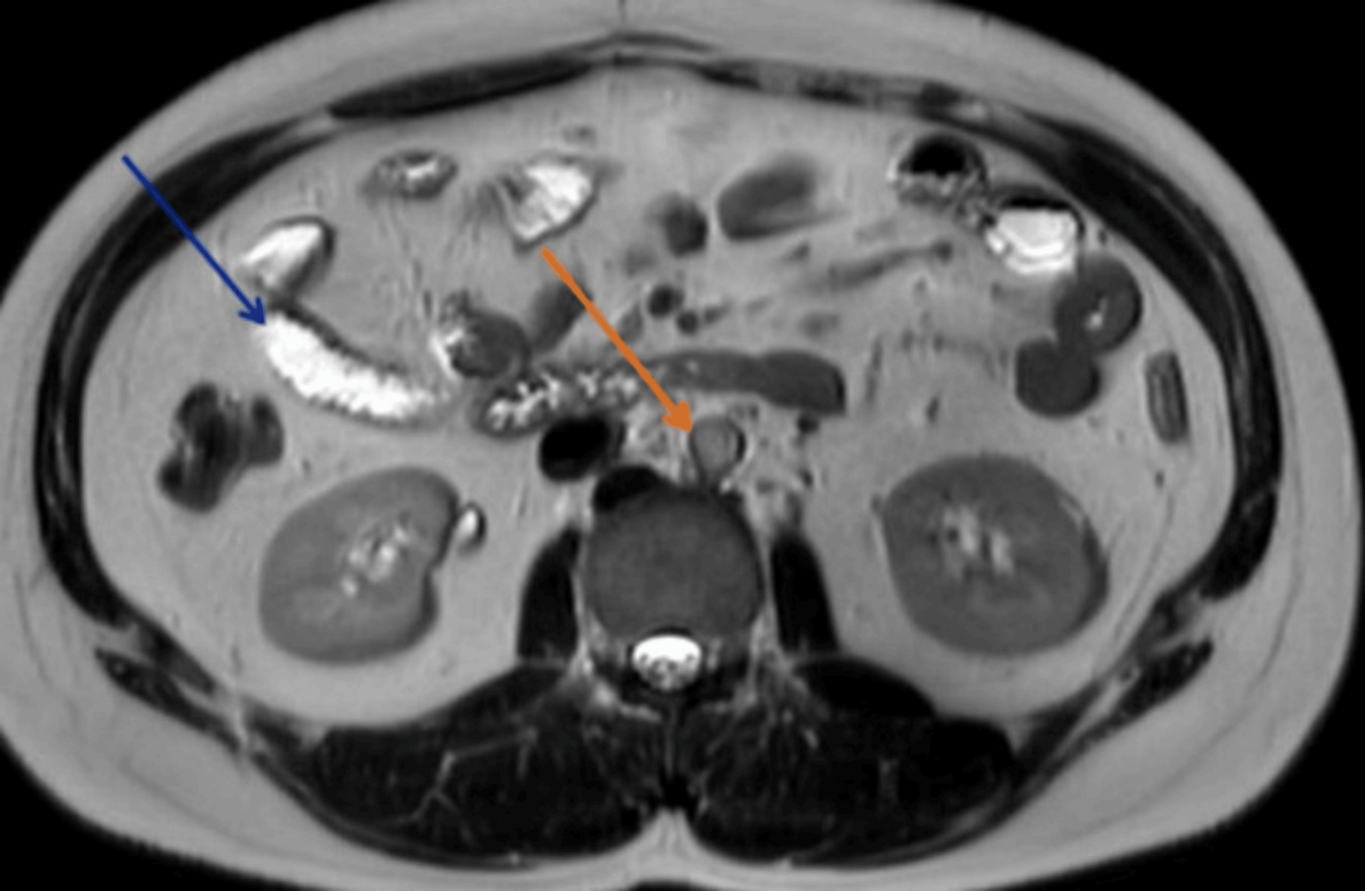
Magnetic resonance cholangiopancreatography An axial T2-weighted MR image shows a conglomerate of necrotic lymph nodes (orange arrow) in the portacaval region, just posterior to the pancreatic head. This nodal mass is causing extrinsic compression and smooth tapering of the distal common bile duct (blue arrow), with resultant upstream biliary dilatation. This appearance is classic for benign extrinsic compression, in this case from tuberculous lymphadenitis.

Given suspicion for distal CBD obstruction, magnetic resonance imaging (MRI) and magnetic resonance cholangiopancreatography (MRCP) were performed and interpreted by a board-certified radiologist with 12 years of experience in abdominal imaging. This revealed a conglomerate of necrotic lymph nodes in the precaval and portacaval region, extending into the pancreatic head and encasing the intrapancreatic portion of the CBD. This resulted in smooth narrowing of the distal CBD with significant upstream biliary dilatation (Figure 2). The largest nodal mass measured 44 × 25 mm. The radiological features, including the conglomerate morphology and necrotic appearance of the nodes, with central low signal on T1 and high signal on T2, were highly suggestive of tuberculous lymphadenitis, making a malignant etiology, such as pancreatic cancer or lymphoma, less likely.

A contrast-enhanced CT scan of the abdomen was also performed, which confirmed conglomerated necrotic lymphadenopathy in the portacaval and precaval regions with central low attenuation (necrosis) and peripheral rim enhancement, a finding characteristic of tuberculous lymphadenitis. No pancreatic mass was identified, and the visualized lung bases showed no pulmonary parenchymal lesions.

MRCP demonstrated smooth tapering of the distal CBD with significant upstream dilatation of the CBD and intrahepatic biliary radicals. The pancreatic duct was normal in caliber, consistent with extrinsic compression without intraluminal pathology.

The patient was admitted for observation and started on intravenous cefuroxime and metronidazole due to a concern for developing cholangitis. Endoscopic retrograde cholangiopancreatography (ERCP) was not performed. The clinical rationale for avoiding ERCP included: (1) the patient had mild cholangitis without systemic features (no fever, hypotension, or organ dysfunction), (2) definitive diagnosis was pursued via endoscopic ultrasound (EUS)-guided biopsy, which also allowed tissue acquisition, (3) EUS-guided fine needle biopsy (EUS-FNB) is preferred over ERCP when tissue diagnosis is needed and cholangitis is mild, and (4) ERCP with stenting is reserved for severe cholangitis, failed medical therapy, or when biliary drainage is urgently needed, consistent with current guidelines.

To obtain a definitive diagnosis, an EUS was performed, and a peripancreatic lymph node biopsy was obtained using a 22-gauge Acquire™ fine needle biopsy (FNB) needle, which provided core tissue for histopathological analysis. The obtained samples were sent for cytology, histopathology, TB polymerase chain reaction (Xpert MTB/RIF Ultra assay), mycobacterial culture, and flow cytometry to rule out lymphoma. Histopathological examination revealed necrotizing granulomatous inflammation with central caseous necrosis, epithelioid histiocytes forming granulomas, multinucleated giant cells (Langhans type), and lymphocytic cuffing. Ziehl-Neelsen staining for acid-fast bacilli (AFB) was negative, which can occur in paucibacillary TB. The Xpert MTB/RIF Ultra assay returned positive for *Mycobacterium tuberculosis* complex DNA, with no rifampicin resistance mutations detected. Mycobacterial culture (MGIT system, Beckton-Dickinson, USA) was sent and remains pending at the time of submission (the eight-week incubation period is not yet complete). Concurrently, an AFB smear and sputum analysis were negative. QuantiFERON-TB Gold Plus assay was performed and returned positive. The patient was also tested for HIV, which was negative. The final diagnosis was obstructive jaundice secondary to tuberculous peripancreatic lymphadenitis with extrinsic compression of the CBD.

Given the significant baseline hepatocellular injury (with alanine aminotransferase (ALT) peaking at 465 U/L, representing >5 times the upper limit of normal), a hepatoprotective anti-tubercular regimen was initiated to minimize the risk of drug-induced liver injury. According to the World Health Organization (WHO) and American Thoracic Society (ATS) guidelines, patients with a baseline ALT >3 times the upper limit of normal should avoid hepatotoxic first-line agents (isoniazid, rifampicin, and pyrazinamide). This decision was made in accordance with guidelines for managing anti-tubercular therapy (ATT) in patients with significant baseline hepatitis or those at high risk of hepatotoxicity. After consultation with the infectious diseases team, the patient was started on moxifloxacin 400 mg daily, ethambutol 1200 mg daily, amikacin 15 mg/kg intravenously (transitioned to intramuscular after two weeks), and linezolid 600 mg daily, along with pyridoxine 50 mg daily. This regimen was chosen due to concerns for hepatotoxicity with standard first-line therapy (isoniazid, rifampicin, and pyrazinamide) in the setting of significant baseline liver dysfunction. A transition to a standard rifampicin-based regimen was planned once liver enzyme levels normalized (ALT <40 U/L). Serial liver function tests showed a progressive downward trend (Table 2). The patient demonstrated significant clinical improvement with resolution of his pain and jaundice within seven days of initiating therapy.

**Table 2:** Trend of key laboratory parameters during hospitalization ALP: Alkaline phosphatase; ALT: Alanine aminotransferase; AST: Aspartate aminotransferase; CRP: C-reactive protein. Note: Reference ranges are based on the institutional standards of Hamad Medical Corporation laboratory.

Date	ALT (U/L)	AST (U/L)	ALP (U/L)	Total bilirubin (μmol/L)	CRP (mg/L)
01/12/25	244	194	317	51	8.1
04/12/25	317	333	250	45	-
05/12/25	465	-	-	38	-
06/12/25	337	150	-	17	7.3
Reference range	0-41 U/L	0-40 U/L	40-130 U/L	<21 μmol/L	<5.0 mg/L

The patient was discharged home on the same regimen after 10 days of hospitalization with close outpatient follow-up. Serial liver function tests at two weeks, four weeks, three months, and six months showed progressive normalization, with all parameters within normal range by three months. Follow-up abdominal ultrasound at three months demonstrated a normalized CBD at 6 mm, persistent but smaller peripancreatic lymph nodes (largest 28 mm), and resolution of gallbladder sludge. Also, follow-up abdominal ultrasound at six months demonstrated a normal CBD (5 mm), significant regression of lymphadenopathy (the largest node now 12 mm), and normal gallbladder appearance. Follow-up MRI at six months confirmed near-complete resolution of the previously observed conglomerate lymphadenopathy with no residual biliary dilatation and no new lesions.

The patient remained clinically well at six-month follow-up, with complete resolution of symptoms and return to normal activities. He will continue ATT for a total of six months (two months of the intensive phase with the current regimen, followed by four months of continuation phase with rifampicin and isoniazid once liver enzymes have fully normalized).

## Discussion

This case demonstrates a rare but clinically important cause of obstructive jaundice. A 29-year-old man from a TB-endemic region presented with painless jaundice and biochemical evidence of biliary obstruction. Initial imaging revealed biliary dilatation without choledocholithiasis or pancreatic mass, prompting further evaluation with MRI, which demonstrated conglomerated necrotic peripancreatic lymphadenopathy causing extrinsic compression of the distal CBD. EUS-guided biopsy with molecular testing confirmed tuberculous lymphadenitis, and the patient was successfully treated with a hepatoprotective anti-tubercular regimen, resulting in complete clinical and biochemical resolution with normalization of imaging at six-month follow-up. This case reports a rare cause of obstructive jaundice - tuberculous peripancreatic lymphadenitis causing extrinsic CBD compression - in a young patient from a TB-endemic region. The diagnostic journey underscores the pivotal role of advanced imaging and tissue acquisition in securing the diagnosis and avoiding unnecessary surgical intervention. Imaging played a crucial role in suggesting the correct etiology. While ultrasonography effectively identified biliary dilatation, it was unable to characterize the underlying cause due to technical limitations and bowel gas interference. MRCP proved invaluable by precisely delineating the extraluminal, compressive nature of the obstruction and effectively ruling out choledocholithiasis. The key imaging features that suggested tuberculous lymphadenitis over malignancy in this case included: (1) conglomerate nodal morphology rather than discrete lymph node enlargement, (2) central necrosis with peripheral rim enhancement on contrast-enhanced CT (hypodense center with enhancing rim), (3) smooth extrinsic compression of the distal CBD rather than irregular infiltrative encasement, (4) absence of a primary pancreatic mass, and (5) the patient's young age [5,6]. These imaging characteristics have been described as classic for tuberculous lymphadenitis in multiple case series [7]. Central necrosis with peripheral rim enhancement is highly suggestive of TB and helps differentiate it from lymphomatous or metastatic lymphadenopathy, which typically demonstrates more homogeneous enhancement [8]. Previous case reports have described similar imaging findings in patients with tuberculous lymphadenopathy causing obstructive jaundice, reinforcing the diagnostic utility of these radiological features [5-7].

Tissue acquisition via EUS-guided biopsy was crucial in securing the definitive diagnosis. EUS-FNB offers several advantages over alternative diagnostic approaches: (1) it provides direct access to peripancreatic and portacaval lymph nodes that may not be accessible via percutaneous approaches, (2) core tissue acquisition (FNB) allows for both cytological and architectural assessment, which is superior to fine needle aspiration (FNA) for diagnosing granulomatous diseases, (3) it avoids the risks associated with ERCP (pancreatitis, perforation, and bleeding) when biliary drainage is not urgently indicated, and (4) it allows for collection of adequate material for molecular testing [2]. The diagnostic yield of EUS-FNA/FNB for tuberculous lymphadenitis has been reported to be 85%-95% in prospective studies [8]. Puri et al. reported a series of 32 patients with mediastinal and abdominal tuberculous lymphadenitis diagnosed by EUS-FNA, demonstrating the procedure's high sensitivity and safety profile [8]. Several case reports have specifically documented the utility of EUS-guided biopsy in diagnosing tuberculous lymphadenopathy presenting with obstructive jaundice [7,8]. In our case, EUS-FNB provided core tissue, revealing necrotizing granulomatous inflammation with caseous necrosis, and the Xpert MTB/RIF Ultra assay confirmed the presence of *M. tuberculosis* complex DNA. The negative AFB smear is not uncommon in paucibacillary TB and does not exclude the diagnosis when molecular testing is positive.

A critical decision point was the choice of ATT in the setting of significant baseline hepatocellular injury. ALT peaking at 465 U/L (>5 times the upper limit of normal), placing him at high risk for drug-induced liver injury from standard first-line ATT [9,10]. According to the ATS, Centers for Disease Control and Prevention (CDC), and Infectious Diseases Society of America (IDSA) guidelines, patients with baseline ALT >3 times the upper limit of normal should receive non-hepatotoxic drug regimens until liver enzymes return to normal [10]. Similarly, the WHO recommends individualized regimen selection for patients with pre-existing liver disease [11]. After consultation with the infectious diseases team, a regimen of moxifloxacin, ethambutol, amikacin, and linezolid was initiated. While linezolid is not typically first-line for drug-susceptible TB, it was selected in this context for its low hepatotoxic potential and was used with infectious disease specialist oversight. Moxifloxacin and linezolid have low hepatotoxic potential, ethambutol is primarily renally cleared with minimal hepatic metabolism, and amikacin has no significant hepatotoxicity. This individualized approach allowed for prompt treatment of TB while mitigating the risk of further hepatic insult. The rapid improvement in liver function tests following initiation of therapy (as shown in Table 2) likely reflects resolution of the obstructive process rather than drug-induced injury, supporting the appropriateness of this regimen.

The decision to avoid ERCP with stenting was also important. Current guidelines from the American Society for Gastrointestinal Endoscopy (ASGE) recommend biliary drainage in the setting of acute cholangitis, particularly in patients with moderate-to-severe disease [9]. However, in patients with mild cholangitis (defined as the absence of organ dysfunction, hypotension, or altered mental status per the Tokyo Guidelines 2018 [9]), a conservative approach with antibiotics and treatment of the underlying cause may be appropriate. Our patient had mild cholangitis (biochemical evidence only, no systemic features), and a definitive diagnosis was pursued via EUS-FNB, which simultaneously provided tissue for diagnosis. This approach avoided the risks of ERCP (5%-10% risk of post-ERCP pancreatitis, 1% risk of perforation, 1%-2% risk of bleeding) while still achieving biliary decompression by treating the underlying lymphadenopathy [9]. The rapid clinical and biochemical improvement following ATT initiation supports the appropriateness of this conservative strategy.

This case has limitations. Mycobacterial culture remains pending at the time of submission, though molecular testing confirmed the diagnosis. Additionally, the unusual ATT regimen, while appropriate in this specific clinical context, may not be generalizable to all patients with TB and liver dysfunction.

## Conclusions

Tuberculous peripancreatic lymphadenitis should be considered in the differential diagnosis of obstructive jaundice, particularly in young patients from TB-endemic regions or when cross-sectional imaging reveals necrotic, conglomerate lymphadenopathy causing extrinsic biliary compression. The imaging features of central necrosis with peripheral rim enhancement on contrast-enhanced CT, along with smooth extrinsic compression of the CBD on MRCP, should raise suspicion for tuberculous etiology and help differentiate this benign condition from malignant causes such as pancreatic cancer or lymphoma. A high index of suspicion, combined with a systematic diagnostic approach using advanced imaging and tissue confirmation via EUS-guided biopsy (with histopathology and molecular testing), is essential to secure the diagnosis. This approach enables the initiation of appropriate, tailored ATT and can prevent patients from undergoing unnecessary and invasive surgical procedures. In patients with significant baseline liver dysfunction, a hepatoprotective ATT regimen selected in consultation with infectious disease specialists should be considered in accordance with international guidelines, with close monitoring of liver function tests. This case adds to the limited literature on tuberculous lymphadenopathy presenting as obstructive jaundice and reinforces the importance of considering TB in the appropriate clinical context.
